# Decoding sialidase: physiological roles, pathological pathways, and clinical opportunities

**DOI:** 10.3389/fcimb.2026.1814721

**Published:** 2026-05-12

**Authors:** Rushi Li, Yafei Liu, Rentong Zou, Meifeng Li

**Affiliations:** 1 The Second Clinical Medical College of Shandong University of Medicine, Yantai, Shandong, China; 2 Department of Critical Care Medicine, Yantai Yuhuangding Hospital Affiliated to Qingdao University, Yantai, Shandong, China

**Keywords:** autoimmune diseases, desialylation, physiological function, sialic acid, sialidase, tumor

## Abstract

This review critically examines pathological dysregulation of sialidase across genetic syndromes, infectious pathogens, inflammatory/autoimmune disorders, malignant transformations, and neurodegenerative cascades, revealing molecular mechanisms that bridge physiological homeostasis to disease pathogenesis. The translational potential is emphasized through its emerging utility as a diagnostic biomarker and therapeutic target, with frank discussion of current challenges in clinical translation-including specificity hurdles, delivery systems, and biomarker validation—and future directions for precision medicine applications. By integrating mechanistic insights with translational strategies, this work provides a roadmap for harnessing sialidase biology to advance prevention, diagnosis, and treatment paradigms for diverse human diseases, offering therapeutic opportunities at the intersection of basic science and clinical innovation.

## 1 Introduction

Recent review articles have substantially advanced the understanding of sialidase biology from several focused perspectives. For example, recent reviews have highlighted the roles of mammalian neuraminidases in immune-mediated diseases, particularly through regulation of mucins, receptor desialylation, and inflammatory signaling, while others have emphasized the contribution of NEU1 to cancer and metabolic disorders or discussed sialyltransferases and neuraminidases as emerging therapeutic targets in oncology (Lillehoj et al., 2022; Nag et al., 2022; Toussaint et al., 2022). These studies have greatly clarified the isoenzyme-specific and disease-specific functions of sialidases. However, a broader synthesis that integrates mammalian and microbial sialidases and links their subcellular localization, substrate specificity, and signaling consequences to pathological pathways and clinical opportunities across multiple disease contexts remains limited. In this review, we therefore provide an updated cross-disciplinary overview of sialidase biology, with particular emphasis on how diverse sialidases contribute to infection, inflammation/autoimmunity, cancer, and neurodegeneration, and how these mechanistic insights may inform biomarker development and therapeutic intervention.

Sialidase, also known as neuraminidase, is a glycoside hydrolase that catalyzes the hydrolysis of α-glycosidic linkages between terminal sialic acid residues and adjacent glycan moieties in glycoconjugates such as glycoproteins and glycolipids (Du et al., 2024; Li et al., 2025). As an important class of enzymes, sialidases participate in diverse physiological processes, including cell adhesion, signal transduction, and immune regulation, and are also implicated in pathological conditions such as infection, inflammation, cancer, and neurodegenerative disease (Lillehoj et al., 2022; Toussaint et al., 2022; Aljohani et al., 2024; Treder and Bączek, 2025). In addition, growing evidence supports their potential value as biomarkers and therapeutic targets (Chen et al., 2024; Schengrund, 2025; van Vliet and van Kooyk, 2025). This review therefore summarizes the classification, structure, biochemical properties, and physiological functions of sialidases, with particular emphasis on their roles in human disease and their diagnostic and therapeutic relevance.

## 2 Fundamental biology of sialidases

### 2.1 Nomenclature and classification of sialidases

Sialidases, also referred to as neuraminidases, are glycosidases that remove terminal sialic acid residues from glycoconjugates (Keil et al., 2022; Alvarado-Melendez et al., 2025). Despite sharing the same biochemical activity, sialidases from mammalian, bacterial, and viral sources exhibit pronounced divergence in protein structure, substrate specificity, subcellular localization, and biological function (Okun et al., 2023; Aljohani et al., 2024; Engibarov et al., 2025). In mammals, four sialidase isoenzymes (NEU1-NEU4) have been identified, each displaying distinct intracellular localization and substrate preference (Okun et al., 2023; Aljohani et al., 2024; Zhu et al., 2024). **Table 1** summarizes their subcellular localization, substrate specificity, and major biological functions. Together, these features underscore the non-redundant roles of mammalian sialidases in lysosomal metabolism, signal transduction, and immune regulation (Lillehoj et al., 2022; Du et al., 2024).

**Table 1:** Subcellular localization, preferred substrates, and major biological functions of human sialidases (NEU1-NEU4).

Sialidase	Major subcellular localization	Preferred substrates	Major biological functions
NEU1 (Miyagi and Yamaguchi, 2012; Lillehoj et al., 2022; Aljohani et al., 2024; Du et al., 2024)	Lysosome, plasma membrane	Sialylated glycoproteins and glycolipids	Lysosomal glycan degradation; regulation of receptor desialylation (e.g., TLRs); control of immune activation and cellular clearance
NEU2 (Oh et al., 2022; Aljohani et al., 2024; Zhu et al., 2024; Huang et al., 2026)	Cytosol	Soluble sialylated glycoconjugates and selected membrane-associated glycoproteins	Regulation of intracellular glycan turnover; implicated in myoblast or neuronal differentiation and apoptosis in specific cellular contexts
NEU3 (Miyagi and Yamamoto, 2022; Aljohani et al., 2024; Tatsuta et al., 2024; Zhu et al., 2024)	Plasma membrane; lipid rafts; extracellular vesicles	Glycosphingolipids, especially gangliosides (e.g., GM3)	Modulation of membrane signaling platforms; activation of EGFR–Akt/ERK pathways via GM3 degradation; regulation of cell proliferation, migration and survival
NEU4 (Yamaguchi et al., 2005; Aljohani et al., 2024; Zhu et al., 2024; Huang et al., 2026)	Lysosomes; mitochondrial membranes;endoplasmic reticulum	Gangliosides (e.g., GD3) and other sialylated glycoconjugates	Regulation of ganglioside homeostasis; modulation of mitochondrial membrane composition; involvement of mitochondria-mediated apoptosis

Microbial sialidases are similarly heterogeneous (Engibarov et al., 2025; Ortega, 2025). Numerous pathogenic bacteria produce one or more sialidases that frequently function as accessory virulence factors by facilitating mucus degradation, host cell adhesion, and tissue invasion, including Clostridium perfringens, which encodes NanH, NanI, and NanJ, and Streptococcus pneumoniae, which expresses multiple neuraminidases (Medley et al., 2024; Engibarov et al., 2025; Gioula and Exindari, 2026). Viral sialidases, most notably the neuraminidase (NA) of influenza A virus, are classified into antigenically distinct subtypes (N1-N9) and play essential roles in viral release, transmission efficiency, and host adaptation (Cardenas et al., 2024; Guo et al., 2024; Gitto et al., 2025; Niu et al., 2025). **Table 2** summarizes the sources and major characteristics of representative microbial sialidases from bacterial and viral pathogens.

**Table 2:** Sources and characteristics of microbial sialidases.

Microorganism	Sialidase/NA type	Localization secretion	Substrate preference	Major biological roles	Contribution to pathogenicity
Clostridium perfringens (Li et al., 2016; Navarro et al., 2018; Navarro et al., 2021; Ba et al., 2024; McClane et al., 2025)	NanH	Mainly intracellular or cell-associated	Sialylated glycoconjugates (general)	Sialic acid scavenging and metabolism	Minor or strain-dependent role in virulence
	NanI	Predominantly secreted (extracellular)	Mucin-associated sialylated glycans	Mucin desialylation; nutrient acquisition	Enhances growth in mucus; promotes intestinal colonization; potentiates toxin (CPE) activity
	NanJ	Secreted or cell-associated (strain-dependent)	Sialylated glycoconjugates	Auxiliary sialidase activity	Accessory role; contributes to colonization in some models
Streptococcus pneumoniae (Gut et al., 2008; Uchiyama et al., 2009; Xu et al., 2011; Montgomery et al., 2025)	NanA	Cell wall-anchored and secreted	Host cell surface sialylated glycans	Exposure of galactose residues; adhesion and invasion	Promotes nasal colonization; epithelial and BBB(Blood Brain Barrier) invasion; biofilm formation
	NanB/NanC	Secreted	Sialylated glycans	Sialic acid acquisition	Support colonization; functional redundancy with NanA
Influenza A virus (Luo et al., 2019; Niu et al., 2025; Kobayashi et al., 2026)	Neuraminidase (NA) N1–N9	Viral envelope glycoprotein	α2, 3- and α2, 6-linked sialic acids	Cleavage of host/viral sialic acids	Essential for virion release, spread, and host adaptation
Trypanosoma cruzi (Nardy et al., 2016; Cruz-Saavedra et al., 2025; dos Santos et al., 2025)	Trans-sialidase (TS)	Secreted and surface-associated	Transfers host sialic acid to parasite surface	Sialic acid acquisition without *de novo* synthesis	Immune evasion; parasite survival and persistence
Neisseria gonorrhoeae (Gulati et al., 2005; Cardenas et al., 2024)	Sialyltransferase	Bacterial surface LOS	Host-derived sialic acid	LOS sialylation	Complement resistance; Siglec engagement

### 2.2 Physiological functions of sialidase

Sialidase, a crucial glycoside hydrolase, is broadly expressed in humans and other mammals as multiple isoenzymes with distinct tissue and subcellular distributions (Aljohani et al., 2024; Du et al., 2024; Schengrund, 2025). Its primary function involves catalyzing the hydrolysis of sialic acid residues at the terminal positions of glycoproteins, glycolipids, and oligosaccharides, and other glycoconjugates (Keil et al., 2022; Zhu et al., 2024). In specific physiological processes, sialidase plays an important regulatory role in cellular recognition, signal transduction, immune modulation, and apoptosis by modifying glycan structures on the cell surface and within the intracellular environment (Lillehoj et al., 2022; Aljohani et al., 2024; Du et al., 2024; Fremuth et al., 2025).

#### 2.2.1 Regulation of glycan modification and its impact on intercellular recognition

**Figure 1:**
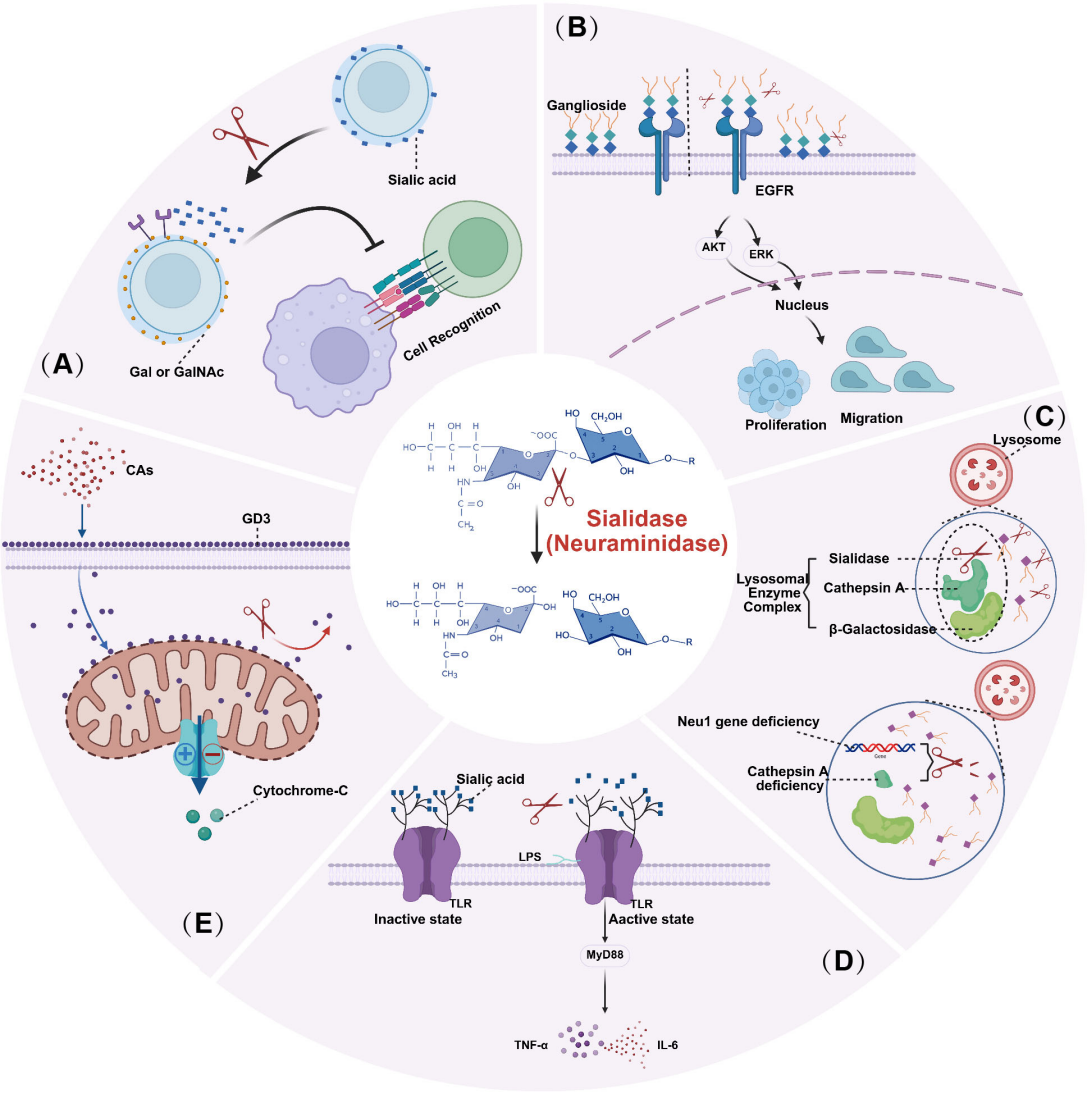
Representative physiological functions of mammalian sialidases. Sialidases regulate diverse cellular processes by removing terminal sialic acid residues from glycoproteins and glycolipids, thereby remodeling glycan-dependent recognition, signaling, lysosomal catabolism, innate immune activation, and cell fate decisions. **(A)** Desialylation of cell-surface glycoconjugates exposes underlying galactose (Gal) or N-acetylgalactosamine (GalNAc) residues, which can alter the binding of lectins and other glycan-recognition molecules and thereby modulate cell-cell recognition, adhesion, and immune interactions. **(B)** At the plasma membrane, NEU3 remodels the ganglioside microenvironment, including the reduction of GM3-related inhibitory constraints on receptor signaling, which can facilitate EGFR activation and downstream AKT/ERK signaling, ultimately promoting cell proliferation and migration. **(C)** In lysosomes, NEU1 functions within a multienzyme complex with cathepsin A and β-galactosidase and is required for the stepwise degradation of sialylated glycoconjugates; NEU1 deficiency disrupts substrate turnover and causes lysosomal accumulation of undegraded material, as occurs in sialidosis. **(D)** During innate immune activation, Neu1 has been proposed to act at Toll-like receptor complexes, where desialylation of receptor ectodomains facilitates receptor activation, adaptor recruitment (e.g., MyD88), and downstream inflammatory signaling, leading to the production of cytokines such as TNF-α and IL-6. **(E)** In overexpression systems, the long isoform of NEU4 has been reported to localize to mitochondria, where it may regulate the sialylation status of mitochondrial membrane gangliosides such as GD3 and thereby influence cytochrome c release and mitochondria-dependent apoptosis. This figure is schematic and summarizes representative mechanisms reported for different mammalian sialidase isoenzymes in distinct subcellular contexts.

Sialic acids are abundantly present at the terminal positions of glycoproteins and glycolipids on the cell membrane, with particularly high distribution in the nervous and immune systems (Zhu et al., 2024; Wang et al., 2025). Sialidase modulates these residues, thereby altering the terminal structure of cell surface glycans and influencing intercellular recognition and adhesion processes (Nardini et al., 2024; Zhu et al., 2024). For instance, as illustrated in **Figure 1A**, sialidase-mediated desialylation exposes galactose (Gal) or N-acetylgalactosamine (GalNAc) residues, which can then serve as ligands for lectins or immune recognition receptors, ultimately affecting cell-cell and cell-matrix interactions (Nardini et al., 2024; Lin et al., 2025; Grosso et al., 2026). Specifically, within the nervous system, sialidase modifies and regulates gangliosides via desialylation, thereby participating in the formation and plasticity of neuronal synapses (Schnaar et al., 2014; Wißfeld et al., 2024). In the immune system, alterations in sialylation status can modulate immune responses by influencing processes such as monocyte chemotaxis and the recognition reactions of natural killer (NK) cells (Lin et al., 2025).

#### 2.2.2 Regulation of cell signal transduction and membrane protein function

Numerous studies have demonstrated that sialidase can significantly influence multiple signaling pathways by removing sialic acid residues from membrane proteins or glycolipids (Keil et al., 2022; Zhu et al., 2024). For instance, NEU3, a member of the sialidase family, is primarily localized to the plasma membrane and exhibits substrate preference for glycosphingolipids such as ganglioside GM3 (Zhu et al., 2024; Jastrząb et al., 2025). The activity of gangliosides is closely associated with the activation of signals including EGFR, Akt, and ERK. Specifically, as shown in **Figure 1B**, plasma membrane-associated NEU3 remodels the ganglioside microenvironment, including GM3-related regulatory constraints on receptor signaling, thereby facilitating EGFR activation and downstream AKT/ERK signaling and ultimately promoting tumor cell proliferation and migration (Zhu et al., 2024; Zeng et al., 2026). Meanwhile, NEU1 can desialylate Toll-like receptor 4 (TLR4) and its co-receptor MD2. This conformational change is a necessary step for TLR4 dimerization, recruitment of downstream adaptor proteins such as MyD88, and subsequent activation of transcription factors including NF-κB, ultimately triggering robust inflammatory responses (Wada et al., 2007; Amith et al., 2009; Bovio et al., 2020).

#### 2.2.3 Involvement in lysosomal function and substrate degradation

As the primary lysosomal sialidase, NEU1 is extensively involved in the degradation of glycoproteins and glycolipids (Itoh and Tsukimoto, 2023). Its activity depends on the formation of a stable complex with cathepsin A (protective protein) and β-galactosidase (Gorelik et al., 2023; Itoh and Tsukimoto, 2023). Deficiency in NEU1 function, as illustrated in **Figure 1C**, can lead to the accumulation of lysosomal carbohydrate substrates, resulting in a rare lysosomal storage disorder known as sialidosis (Li et al., 2024; Peng et al., 2025). Clinically, this condition manifests as progressive neurodegenerative changes and hepatosplenomegaly, among other symptoms (Ding et al., 2024; Kılıç et al., 2025). Therefore, NEU1 plays a crucial role in maintaining intracellular metabolic homeostasis (Abdulkhalek et al., 2011; Itoh and Tsukimoto, 2023).

#### 2.2.4 Modulation of immune response and cell clearance

Sialidase precisely regulates immune responses through desialylation. For example, as shown in **Figure 1D**, pathogen-associated molecular patterns (PAMPs, TLR ligands) do not simply bind directly to their receptors. Instead, they induce the activation of Neu1 at the receptor complex. By removing sialic acid residues from the extracellular domains of receptors (desialylation), Neu1 promotes receptor conformational changes and signal transduction, thereby modulating the immune response (de Geest et al., 2002; Amith et al., 2009). Furthermore, scholars have proposed that sialic acid-rich glycan structures on the surface of healthy cells function as “self-associated molecular patterns (SAMPs), “ allowing them to evade immune attack. This makes the desialylation activity of sialidase even more critical for precise immune regulation (Varki, 2011). In the case of immune thrombocytopenia (ITP), studies have found that the removal of sialic acid from platelet surfaces by sialidase exposes galactose residues. These exposed residues are recognized by the Ashwell-Morell receptor on hepatocytes, leading to platelet clearance (Li et al., 2015).

#### 2.2.5 Regulation of apoptosis and autophagy

Recent studies have revealed that members of the sialidase family play distinct roles in the regulation of cell death processes (Keil et al., 2022; Zhu et al., 2024). For instance, as shown in **Figure 1E**, NEU4 (the long isoform) in overexpression systems can localize to mitochondria and modulate the sialylation status of mitochondrial membrane gangliosides, such as GD3, thereby influencing cytochrome c release mitochondrial-mediated apoptosis (Yamaguchi et al., 2005; Bourguet et al., 2022). Meanwhile, NEU3 has been reported to regulate the metabolism of gangliosides and ceramide at the plasma membrane and intracellularly, indirectly affecting cell apoptosis and survival (Schengrund, 2025). However, these mechanisms exhibit heterogeneity across different cell types and experimental conditions. Many conclusions are still based on studies involving overexpression or knockdown, and thus the endogenous regulatory mechanisms require further validation (Hasegawa et al., 2007; Giussani et al., 2014; Keil et al., 2022).

In summary, sialidases not only play a crucial role in glycan modification but are also central to a multitude of physiological functions, including cell recognition, signal regulation, immune modulation, substrate degradation, and cell fate determination. Due to their differences in spatial distribution, enzymatic specificity, and regulatory mechanisms, sialidases assume distinct physiological roles in various tissues and cellular states. Consequently, their dysregulation is often closely associated with the pathogenesis of diverse diseases, which lays a solid foundation for the subsequent discussion on their pathological mechanisms and potential as therapeutic targets.

## 3 Role of sialidases in human diseases

Sialidase plays a pivotal regulatory role in numerous physiological processes. Its aberrant expression or functional dysregulation is now recognized as being closely associated with the pathogenesis of various human diseases. By cleaving terminal sialic acid residues from glycoproteins, glycolipids, and oligosaccharides, sialidase influences a wide array of cellular events, including signal transduction, cell recognition, and adhesion. Furthermore, it serves a crucial function in the modulation of inflammation, immune responses, metabolic homeostasis, and cell fate decisions. For instance, in recent years, the pathogenic mechanisms involving sialidase have demonstrated significant relevance in hereditary disorders, infectious diseases, inflammatory and autoimmune conditions, malignant tumors, and neurodegenerative diseases, findings that have been substantiated across multiple disease models.

### 3.1 Hereditary diseases: metabolic disorders caused by sialidase deficiency

In humans, mutations in the NEU1 gene are the direct cause of the most common sialidase-related inherited disorder: sialidosis (SiD) (Li et al., 2024; Peng et al., 2025). As NEU1 is primarily localized to lysosomes, and its proper localization, stability, and catalytic activity within lysosomes largely depend on its incorporation into the lysosomal multienzyme complex (LMC) formed with the protective protein cathepsin A (also known as PPCA, encoded by the CTSA gene), mutations in the NEU1 gene or deficiency of CTSA often lead to a significant loss of NEU1 activity or its instability (Caciotti et al., 2013; Gorelik et al., 2021; Itoh and Tsukimoto, 2023). Specifically, NEU1 deficiency results in the abnormal accumulation of sialylated glycoconjugates (such as sialylglycopeptides and sialyloligosaccharides) within lysosomes of neural and visceral tissues (Bonten et al., 2000; Peng et al., 2025). This accumulated substrate triggers lysosomal swelling, vacuolization, and disruption of cellular homeostasis, clinically manifesting as neurological involvement (e.g., myoclonus/epilepsy, ataxia, corneal clouding, and macular cherry-red spot) and visceral involvement (e.g., hepatosplenomegaly) (Annunziata and d’Azzo, 2017). Currently, two distinct diseases are strongly associated with these defects: Sialidosis (SiD), arising directly from NEU1 enzyme deficiency due to mutations in the NEU1 gene; and Galactosialidosis (GS), resulting indirectly from CTSA gene mutations leading to combined loss of NEU1 and partial GLB1 activity (while these disorders share biochemical and clinical features, their pathogenic mechanisms differ) (Caciotti et al., 2013; Peng et al., 2025).

### 3.2 Infectious diseases: sialidases facilitate pathogen invasion and spread

#### 3.2.1 Mechanisms of disrupting host mucus and epithelial barriers

**Figure 2:**
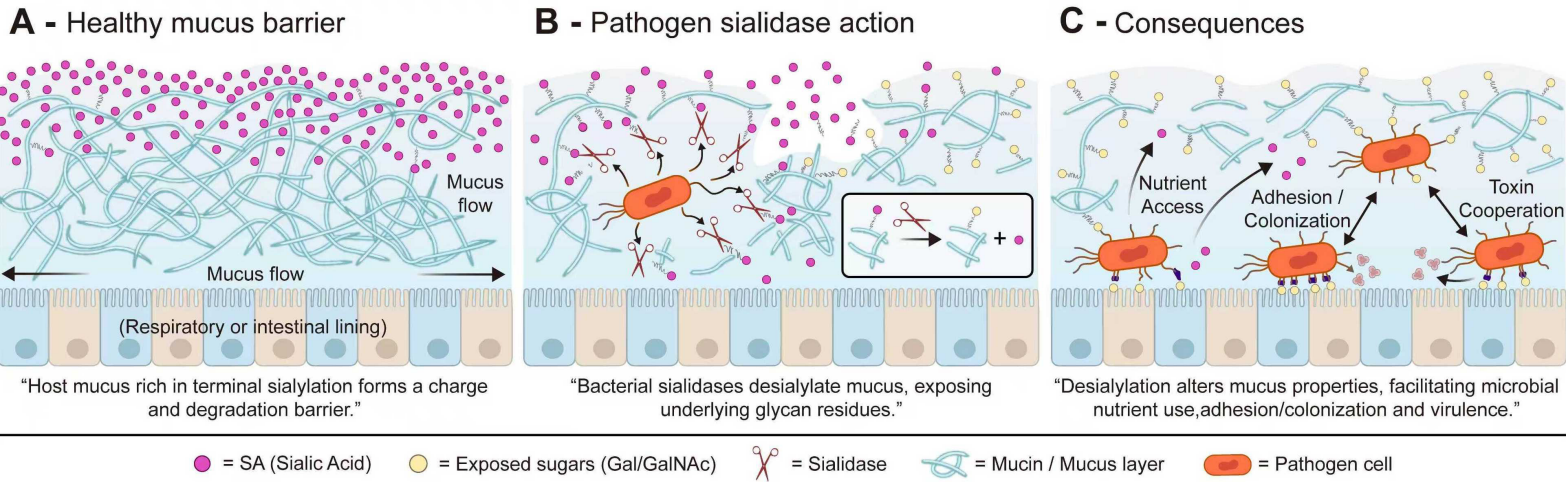
Pathogen sialidase-mediated disruption of the mucus barrier promotes nutrient access, adhesion, colonization, and virulence. **(A)** In healthy respiratory or intestinal mucosa, mucins are densely decorated with terminal sialic acids (SA), which help maintain a hydrated, negatively charged, and relatively degradation-resistant mucus barrier. This barrier limits microbial penetration and reduces direct access of pathogens to the epithelial surface. **(B)** Pathogen-derived sialidases cleave terminal SA residues from mucin glycans, thereby releasing sialic acids and exposing underlying galactose (Gal) or N-acetylgalactosamine (GalNAc) residues. This desialylation weakens the protective physicochemical properties of mucus and can facilitate further degradation of mucin glycans by other microbial enzymes. **(C)** As a consequence, the altered mucus layer becomes more permissive to infection: liberated or exposed glycans may support microbial nutrient acquisition, exposed subterminal residues may enhance pathogen adhesion and colonization, and the weakened mucus barrier may promote synergistic action with toxins or other virulence factors, thereby increasing overall pathogenicity.

Mucins secreted by epithelial surfaces in the respiratory and intestinal tracts are heavily decorated with terminally sialylated glycans, and this terminal sialylation is important for maintaining mucus barrier integrity by preserving the negative charge, network architecture, and resistance of mucins to excessive bacterial degradation and invasion (Yao et al., 2022; Taniguchi et al., 2023). By desialylating these structures, sialidases alter the surface properties of mucus and facilitate further degradation by other glycosidases, thereby compromising the integrity of the mucosal barrier (Shuoker et al., 2023; Medley et al., 2024). As shown in **Figure 2**, bacterial sialidases from intestinal and respiratory pathogens cleave terminal sialic acids from mucins, weakening the protective mucus barrier and exposing underlying glycans, thereby promoting microbial nutrient access, adhesion/colonization, and virulence enhancement (Navarro et al., 2021; Yao et al., 2022; Montgomery et al., 2025). Taking Clostridium perfringens as an example, this species produces three bacterial sialidases: NanH, NanI, and NanJ. Among these, NanI plays a predominant role and has been demonstrated to enhance the growth and attachment of C. perfringens in the presence of mucus, as well as potentiate the pathogenic effects of certain toxins (e.g., CPE) (Wang, 2020; Li et al., 2021; Navarro et al., 2021). However, the role of sialidase is not consistent or strictly “essential” across different bacterial strains, host tissues, or animal models. Current research indicates that deletion of nanI significantly impairs the colonization or virulence of C. perfringens in intestinal mucus-associated models (Chiarezza et al., 2009). Conversely, in certain models of myonecrosis, individual deletion of nanI or nanJ, or deletion of both, does not markedly attenuate pathogenicity, suggesting that the contribution of sialidase to the disease process is, to some extent, strain- and model-dependent (Chiarezza et al., 2009). In summary, a more cautious conclusion is that sialidase typically acts as a promotive or auxiliary factor. It assists microorganisms in breaching the mucosal barrier through desialylation and can enhance colonization and certain virulence mechanisms, but it is not indispensable in all infection scenarios.

#### 3.2.2 Exposure of underlying glycan residues to enhance adhesion and invasion capability

The desialylation activity of sialidase not only removes terminal sialic acids from host surfaces but also exposes underlying glycan motifs, particularly galactose-containing and N-acetylglucosamine/lactosamine-related residues (Mathew et al., 2023). Taking *Streptococcus pneumoniae* as an example, its surface sialidase NanA removes sialic acids from host cells, particularly epithelial or cerebrovascular endothelial cells, exposing underlying galactose residues (Montgomery et al., 2025). In certain pathogen-host interactions, these exposed residues can be recognized by bacterial adhesins, thereby enhancing adhesion or invasion (Singh et al., 2014). Studies on *S. pneumoniae* have shown that a NanA-deficient strain (ΔNanA) exhibits significantly reduced capabilities in nasal colonization, mucosal invasion, and biofilm formation in *in vivo* models, indicating that NanA plays an important role in promoting bacterial colonization via desialylation (Blanchette et al., 2016). In models using human brain microvascular endothelial cells (hBMECs), NanA is a necessary factor for promoting the adhesion and invasion of *S. pneumoniae*: for instance, the ΔNanA mutant shows markedly decreased adhesion and invasion, while exogenous expression of NanA in other strains or genetic complementation can partially restore this ability (Uchiyama et al., 2009). Furthermore, the sequence and form of NanA exhibit considerable diversity among different strains (including variants such as “cell wall-anchored” and “secreted” forms), and these variations can influence its enzymatic properties and pathogenic manifestations to some extent (Xu et al., 2016; Gratz et al., 2017). In summary, the role of NanA in promoting attachment, colonization, and invasion is robust and supported by multiple studies; however, its specific contribution is dependent on the bacterial strain, host tissue, and animal model. In some contexts, its absence significantly reduces pathogenicity, while in others, or when complemented by other virulence factors, its impact may be diminished or partially compensated.

#### 3.2.3 Immune evasion: glycan “camouflage” and host immunosuppression

**Figure 3:**
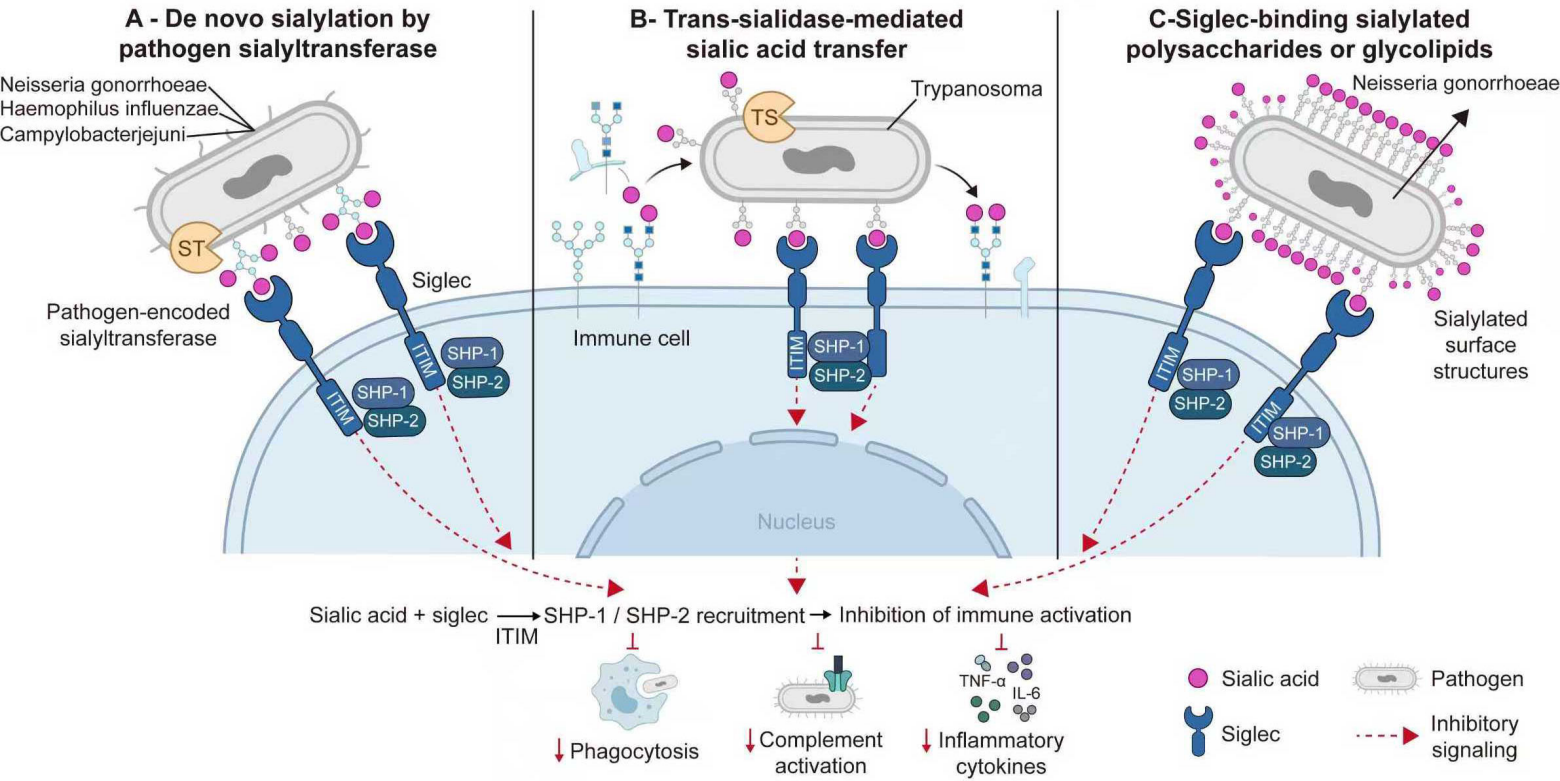
Pathogen immune evasion via sialic acid-mediated engagement of inhibitory siglec receptors. Terminal sialic acids on host cell surface and plasma glycoconjugates function as self-associated molecular patterns that are recognized by inhibitory Siglec receptors on innate immune cells. Several pathogens exploit this host glyco-immune checkpoint through molecular mimicry. **(A)** Some bacteria, including Neisseria gonorrhoeae, Haemophilus influenzae, and Campylobacter jejuni, express pathogen-encoded sialyltransferases that add sialic acids to surface glycans such as LOS or capsular polysaccharides, thereby generating host-like sialylation patterns. **(B)** Other pathogens, such as Trypanosoma spp., express trans-sialidases that transfer sialic acids from host glycoconjugates to the pathogen surface, forming a “theft-and-transfer” camouflage mechanism. **(C)** In addition, some pathogens maintain or modify sialylated polysaccharides or glycolipids that directly engage inhibitory Siglec receptors on host immune cells. Upon Siglec ligation, immunoreceptor tyrosine-based inhibitory motif (ITIM)-dependent signaling recruits SHP-1 and SHP-2 phosphatases, resulting in inhibition of immune cell activation, reduced phagocytosis, decreased complement activation, and lower inflammatory cytokine production, thereby promoting pathogen survival and persistence.

Terminal sialic acids on host cell surfaces and plasma proteins can function as self-associated molecular patterns that dampen immune responses through engagement of inhibitory Siglec receptors (Coccimiglio et al., 2024; Feng et al., 2024). Consequently, some pathogens have evolved various strategies to exploit or mimic this “sialic acid camouflage” to evade immune surveillance (de Jong et al., 2022; Alvarado-Melendez et al., 2024; Guerrero-Flores et al., 2025). As illustrated in **Figure 3**, these mechanisms can be broadly divided into three categories.

First, pathogens can express their own sialyltransferases (STs) to incorporate sialic acids onto their surfaces (Alvarado-Melendez et al., 2024; Dhanabalan et al., 2024). For example, pathogens such as *Neisseria gonorrhoeae*, *Haemophilus influenzae*, and *Campylobacter jejuni* can express their own sialyltransferases to add sialic acids to lipooligosaccharides (LOS) or surface polysaccharides (Gill et al., 1996; Hood et al., 1999; Guerry et al., 2000; Jackson et al., 2021; Cardenas et al., 2024; Omole et al., 2024). By mimicking host surface glycans through sialylation, they can inhibit complement activation, engage Siglec receptors, or interfere with antibody recognition, thereby promoting immune evasion and, in some cases, enhancing colonization or persistence (de Jong et al., 2022).

Second, pathogens can utilize trans-sialidases to transfer sialic acids from the host onto their own surfaces, i.e., a “theft/transfer” mechanism (Wang et al., 2025). For instance, *Trypanosoma* species express a trans-sialidase (TS) that transfers sialic acids from host glycans to the parasite surface, thereby modulating parasite-host interactions, facilitating immune evasion, and supporting persistent survival within the mammalian host (Cruz-Saavedra et al., 2025; dos Santos et al., 2025).

Third, some pathogens can maintain or modify sialylated polysaccharides or glycolipids that directly engage inhibitory Siglec receptors on host immune cells (Chang and Nizet, 2020; de Jong et al., 2022). Upon Siglec ligation, immunoreceptor tyrosine-based inhibitory motif (ITIM)-dependent signaling is activated, with subsequent recruitment of SHP-1 and SHP-2 phosphatases, thereby suppressing immune cell activation, phagocytosis, complement activation, and inflammatory cytokine production, ultimately promoting pathogen survival and immune escape (Cardenas et al., 2024; Schaapherder et al., 2025). For example, sialylated surface structures (e.g., capsule or LOS) of *Neisseria meningitidis* can contribute to complement resistance and may also interact with inhibitory Siglec receptors, thereby attenuating immune clearance and facilitating evasion of host immunity (Jarvis and Vedros, 1987; Lewis et al., 2012; Chang and Nizet, 2020; Di Carluccio et al., 2025).It is important to emphasize that these mechanisms vary among different pathogens, hosts, and tissues; thus, the contribution of trans-sialidase or sialyltransferase to immune evasion, while widespread, remains context-dependent.

#### 3.2.4 Facilitating the action of toxins or other pathogenic factors

Sialidases (neuraminidases) expressed by certain pathogens not only alter host cell surface architecture by removing terminal sialic acids from host glycans but also enhance pathogenicity by facilitating the catalytic activity or potency of toxins and other virulence factors (Navarro et al., 2021; Shizukuishi et al., 2024; Shrestha et al., 2024). For instance, the sialidase NanA of *Streptococcus pneumoniae* not only promotes adhesion but may also fine-tune pneumolysin-mediated membrane disruption by trimming sialic acids from host membrane-associated glycans, thereby optimizing intracellular bacterial survival (Shizukuishi et al., 2024; Fritsch et al., 2025). Furthermore, in the mucus-covered environment of the gastrointestinal tract, the sialidase NanI of *Clostridium perfringens* significantly potentiates the pathogenic activity of its major toxin, CPE, by increasing toxin binding and pore formation and aggravating epithelial injury and barrier dysfunction (Navarro et al., 2021). In this context, sialidase NanI functions as an accessory virulence factor (Navarro et al., 2021; Shrestha et al., 2024). In summary, the role of sialidase in infectious diseases extends beyond its fundamental function of “removing host sialic acid modifications.” It also acts as a cooperative factor for pathogens by reshaping the host glycan environment to improve the binding efficiency and penetration capability of toxins and other virulence factors, thereby amplifying overall pathogenicity (Navarro et al., 2021; Shizukuishi et al., 2024; Shrestha et al., 2024).

#### 3.2.5 Promoting viral release and spread

**Figure 4:**
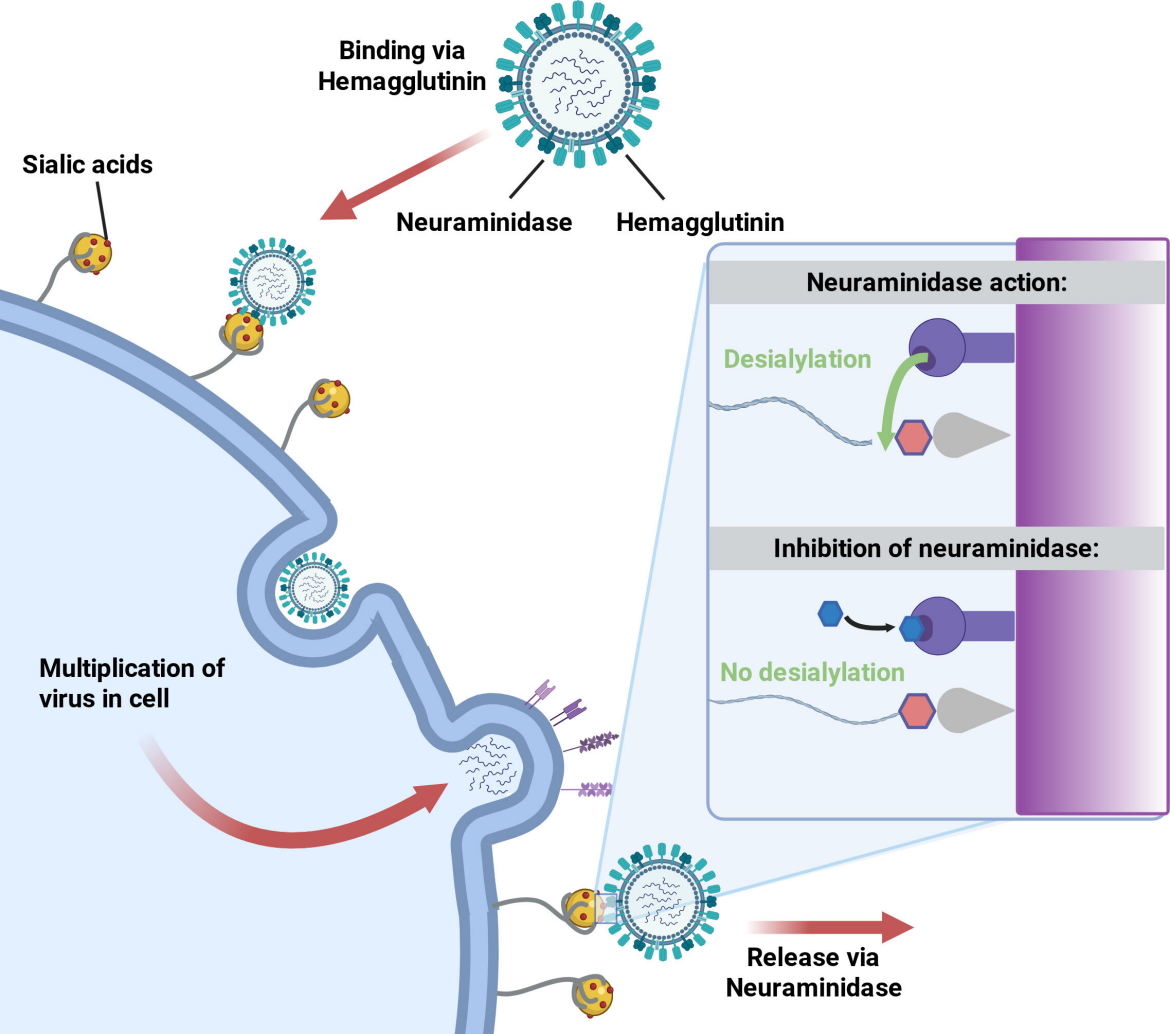
Neuraminidase facilitates influenza virus release and propagation by removing terminal sialic acids. Hemagglutinin (HA) mediates the initial attachment of influenza virions to sialic acid-containing receptors on the host cell surface, enabling viral entry. After intracellular replication and budding, newly formed virions remain susceptible to HA-mediated reattachment to sialylated receptors on infected cells, neighboring cells, mucus components, or other virions. Neuraminidase (NA) cleaves terminal sialic acids from these cellular and extracellular glycoconjugates, thereby preventing virion aggregation and release blockade and promoting efficient progeny virus dissemination. The inset illustrates that, when NA activity is inhibited, desialylation does not occur, and virions remain tethered to sialylated receptors, resulting in impaired viral release.

In certain enveloped viruses such as influenza viruses, neuraminidase (NA) plays a crucial role. As illustrated in **Figure 4**, hemagglutinin (HA) first mediates viral attachment to sialic acid-containing receptors on the host cell surface, whereas after viral replication and budding, NA promotes efficient progeny virion release by cleaving terminal sialic acids from infected cells, neighboring cell surfaces, mucus components, and newly formed viral particles (Palese et al., 1974; Cohen et al., 2013). This receptor-destroying activity prevents HA-mediated virion aggregation and reattachment to cellular or decoy receptors, thereby facilitating viral dissemination. Conversely, inhibition of NA blocks desialylation, causing newly formed virions to remain tethered to sialylated surfaces and impairing viral release (Palese et al., 1974; Cohen et al., 2013; Iseli et al., 2023). The functional balance between neuraminidase activity and hemagglutinin receptor-binding properties is essential for viral fitness and transmissibility (Wallace et al., 2023; Thompson et al., 2024). For instance, variations in the sialic acid environment of different host airways can drive the functional co-evolution of HA and NA, thereby influencing the efficiency of cross-species transmission (Mitnaul et al., 2000; Iseli et al., 2023). Moreover, other respiratory viruses also employ analogous receptor-destroying proteins: certain paramyxoviruses use a hemagglutinin-neuraminidase (HN) protein, some embecoviruses encode hemagglutinin-esterase (HE), and influenza C/D viruses possess hemagglutinin-esterase-fusion (HEF) proteins (Lang et al., 2020; Naveed et al., 2024; Wu et al., 2024). These proteins promotes viral release and dissemination by coordinating receptor binding and receptor-destroying activity.

### 3.3 Inflammatory and autoimmune diseases: sialidase-mediated immune activation and excessive response

Sialidases (neuraminidases, NEUs) modulate immune signaling, membrane receptor stability, and protein degradation by removing sialic acid residues from cell surfaces and secreted proteins (Aljohani et al., 2024; Du et al., 2024; Jia et al., 2024). In specific pathological contexts, this process can contribute to the amplification of inflammation, the promotion of autoimmune responses, and induction of tissue damage (Heimerl et al., 2022; Min et al., 2025). For instance, desialylation can attenuate inhibitory sialoglycan-mediated “self” signals, thereby lowering the threshold for immune activation, and may promote chronic inflammatory or autoimmune processes by altering adhesion- and clearance-related pathways, including macrophage uptake of desialylated ligands (Demina et al., 2021; Lillehoj et al., 2022; Lin et al., 2025).

#### 3.3.1 Airway inflammation and asthma: the NEU1-CD44 axis drives T helper 2 responses

Studies have shown that NEU1 is dysregulated and often upregulated in airway tissues and structural cells in asthma (Mei et al., 2024). T helper 2 (Th2) cells are a subset of CD4+ T lymphocytes that characteristically produce type 2 cytokines, including IL-4, IL-5, and IL-13, and play a central role in allergic airway inflammation (Harker and Lloyd, 2023; Xie et al., 2024). Specifically, NEU1-mediated desialylation of CD44 enhances its binding affinity to hyaluronic acid (HA), thereby promoting the retention of antigen-specific Th2 cells in the airways and amplifying inflammatory responses (Katoh, 2021; Lillehoj et al., 2022). Moreover, inhibition or genetic deficiency of NEU1 attenuates airway hyperresponsiveness and inflammatory cell infiltration, indicating that NEU1 is an important mediator of asthma-associated airway inflammation and remodeling (Katoh, 2021; Mei et al., 2024).

#### 3.3.2 Intestinal inflammation and ulcerative colitis: the NEU3-IAP pathway disrupts mucosal immune balance

Research indicates that in a mouse model of colitis induced by repeated infection with the foodborne pathogen *Salmonella* Typhimurium, the expression of NEU3 in the intestinal epithelium is significantly upregulated, concomitant with the activation of TLR4 signaling (Yang et al., 2021; Crouch et al., 2024). Specifically, NEU3-mediated desialylation of intestinal alkaline phosphatase (IAP) shortens its half-life and reduces its stability and anti-inflammatory function (Lallès, 2014; Yang et al., 2021). This leads to the accumulation of LPS-P, impairment of the intestinal barrier, and amplification of inflammation. The finding that either knocking out NEU3 or supplementing with IAP significantly alleviates inflammation and tissue damage reveals NEU3 as a key pathogenic factor that disrupts intestinal homeostasis and promotes inflammatory responses (Yang et al., 2021; Ma et al., 2024).

#### 3.3.3 Immune thrombocytopenia: desialylation promotes liver AMR-mediated platelet clearance

In some patients with immune thrombocytopenia (ITP), particularly those positive for anti-GPIbα antibodies, autoantibodies can induce the desialylation of platelet surfaces, exposing terminal galactose residues (Li et al., 2015; Zheng et al., 2022; Zheng and Perdomo, 2024). These desialylated platelets are subsequently recognized and cleared by the Ashwell-Morell receptor (AMR, also known as ASGPR) on hepatocytes, leading to thrombocytopenia (Li et al., 2015; Chen et al., 2022). The extent of platelet desialylation may also correlate to some degree with disease activity and therapeutic response (Li et al., 2015). Furthermore, animal studies have demonstrated that blocking AMR or administering neuraminidase inhibitors (such as oseltamivir) can partially restore platelet counts, suggesting that the desialylation-AMR pathway may represent a novel therapeutic target for ITP (Li et al., 2015; Qiu et al., 2016).

#### 3.3.4 Rheumatoid arthritis: EPO-JAK2/STAT5-NEU3 signaling drives synovial fibroblast activation

Recent studies have demonstrated that erythropoietin (EPO) activates the JAK2/STAT5 signaling pathway in fibroblast-like synoviocytes (FLS) via its receptor EPOR, thereby promoting STAT5 binding to the NEU3 promoter and upregulating NEU3 transcriptional expression (Emori et al., 2020; Wu et al., 2024). Within this context, it was found that NEU3 overexpression enhances the desialylation degree on the FLS surface, subsequently increasing their migration and invasion capabilities (Wu et al., 2024). This mechanism is believed to accelerate joint destruction and disease progression in rheumatoid arthritis (Emori et al., 2020; Wu et al., 2024). Conversely, inhibition or knockdown of NEU3 significantly attenuates the invasiveness of FLS and partially mitigates joint damage in rheumatoid arthritis models (Wu et al., 2024).

### 3.4 Malignant tumors: sialidases remodel tumor glycosylation and signaling pathways

As a terminal modification of glycan chains, sialic acid is significantly enriched on the surface of various tumor cells. Its dynamic equilibrium is co-maintained by sialyltransferases and sialidases (human NEU1-NEU4). In recent years, substantial evidence indicates that sialidases participate in tumor proliferation, apoptosis regulation, migration/invasion, immune evasion, and therapy resistance through specific desialylation activities (Dobie and Skropeta, 2021; Nag et al., 2022; Toussaint et al., 2022; Gorelik et al., 2023). Overall, NEU3 (primarily plasma membrane-associated, with a preference for ganglioside substrates) and NEU1 (predominantly lysosomal, but capable of appearing at the cell surface under specific conditions and requiring association with protective protein/cathepsin A [CTSA] within the lysosomal multienzyme complex for proper stability and catalytic activity)are considered to play significant roles in various cancer contexts (Mozzi et al., 2015; Toussaint et al., 2022; Gorelik et al., 2023; Itoh and Tsukimoto, 2023). The specific mechanisms primarily encompass the following aspects.

#### 3.4.1 NEU3 regulates receptor tyrosine kinase and lipid raft functions by remodeling membrane ganglioside profiles

NEU3, a plasma membrane-associated and ganglioside-specific sialidase, alters the composition and aggregation state of glycolipids and proteins within lipid rafts by catalyzing the desialylation of gangliosides such as GM3 (Cirillo et al., 2016). Specifically, in various tumor cell lines and animal models, NEU3 reduces GM3 levels and remodels the lipid raft microenvironment through desialylation of membrane-associated GM3. This action attenuates the inhibitory effect of GM3 on receptor tyrosine kinases like EGFR, thereby promoting receptor clustering and activation. Consequently, it enhances the activity of downstream pro-proliferative and anti-apoptotic signaling pathways such as Ras-ERK and PI3K-Akt (primarily evidenced by *in vitro*mechanisms) (Kawamura et al., 2012; Mozzi et al., 2015). Furthermore, in *in vitro* models of renal cell carcinoma, NEU3 has been shown to promote cancer cell migration and invasion by modulating the endocytosis, recycling, and focal adhesion signaling of the cell surface receptor β1-integrin (cellular/molecular evidence) (Tringali et al., 2012). Current studies on tumor tissues and functional assays indicate that NEU3 expression is upregulated in several solid tumors (e.g., head and neck squamous cell carcinoma, bladder cancer, gastric cancer). Inhibition of NEU3 in head and neck squamous cell carcinoma and bladder cancer effectively reduces pro-growth signals such as ERK/PI3K, thereby impairing cell migration and invasion capabilities (Shiga et al., 2015; Quirino et al., 2022; Tatsuta et al., 2024).

In summary, these findings suggest a pro-tumorigenic role for NEU3 in certain cancer types, highlighting its potential as a therapeutic target. However, it is important to emphasize that these conclusions are primarily based on *in vitro*models, animal studies, and limited clinical cohorts. Given the heterogeneous role of NEU3 across different tumor types, validation through larger-scale patient cohorts and translational research remains necessary.

#### 3.4.2 Sialidase-mediated glycan remodeling promotes tumor invasion

Studies indicate that sialidases, particularly NEU1 and NEU3, can remodel the glycans on specific cell surface molecules such as β1-integrin and CD44 through desialylation. This remodeling affects their adhesion capacity, endocytosis and recycling efficiency, and effectively modulates associated adhesion-signaling axes (e.g., FAK/AKT). Consequently, these structural and functional alterations are linked to enhanced cell migratory properties (Katoh et al., 2010; Tringali et al., 2012; Mozzi et al., 2015). Specifically, NEU3 can effectively promote cell migration and invasion by regulating the endocytosis and recycling of β1-integrin, activating FAK signaling, and enhancing the EGFR–ERK/Akt pathway, among other mechanisms (Tringali et al., 2012). Furthermore, the desialylation-mediated remodeling of CD44 glycosylation by sialidases enhances its binding to hyaluronic acid (HA) (Katoh et al., 2010). Additionally, alterations in glycosylation, including sialylation, may modulate the interactions of CD44 with mucins (MUC) and fibronectin (Liao et al., 2022). Collectively, these changes not only strengthen cell-matrix adhesion, signal transduction, and migratory potential but also theoretically facilitate the establishment of more stable attachments by tumor cells at the invasive front, thereby further promoting invasion. It should be noted that as most of these conclusions are derived from *in vitro* mechanistic studies or analyses in mice and histology, and given the heterogeneity among different tumor types and clinical cohorts, caution is warranted when interpreting their potential for clinical translation.

#### 3.4.3 Altered sialidase profiles and immune evasion

Tumor cells frequently exhibit a phenomenon known as “hypersialylation, “ primarily driven by the upregulation of sialyltransferases (STs). This hypersialylation promotes immune evasion by engaging inhibitory Siglec receptors on the surface of immune cells. The interaction triggers ITIM-mediated inhibitory signaling, thereby suppressing the effector functions of NK cells, macrophages, and T cells (Dobie and Skropeta, 2021; Läubli et al., 2022). Specifically, as neuraminidases/sialidases dynamically modulate the types, distribution, and accessibility of sialic acids in the tumor microenvironment, they can exert dual regulatory effects. In some contexts, desialylation reduces the availability of ligands recognized by Siglecs, thereby attenuating inhibitory signals. In other scenarios, desialylation may expose specific structures that favor Siglec recognition, potentially enhancing inhibitory signaling (Macauley et al., 2014; Nag et al., 2022; Stanczak and Läubli, 2023; Aljohani et al., 2024). In summary, while hypersialylation is a major driver of tumor-associated immunosuppression, sialidases may play a nuanced, fine-tuning role in tumor immunology by altering the spatial and chemical characteristics of the sialic acid landscape (Nag et al., 2022; Stanczak and Läubli, 2023).

#### 3.4.4 Sialidases and tumor therapy sensitivity

Sialidases (neuraminidases) can significantly impact the sensitivity of tumors to chemotherapy, targeted therapy, and immunotherapy by reprogramming the glycan composition of cancer cells and the tumor microenvironment (Pinho et al., 2025; van Vliet and van Kooyk, 2025; Bashian et al., 2026). Specifically, their effects involve molecular, cellular, and immunological dimensions.

Firstly, NEU1 can desialylate EGFR, thereby promoting receptor dimerization and activation, which enhances downstream PI3K/Akt and Ras/ERK signaling and may contribute to tumor cell resistance to anticancer therapy (Du et al., 2024; Jastrząb et al., 2025). For instance, inhibiting NEU1 can, to some extent, reverse drug resistance and restore tumor cell sensitivity to chemotherapy, as evidenced by *in vitro* studies and mouse models (O’Shea et al., 2014).

Secondly, tumor cells often exploit high-level sialylation (hypersialylation) to engage inhibitory Siglec receptors, such as Siglec-7, Siglec-9, and Siglec-E, on immune cells including NK cells and macrophages, thereby transmitting inhibitory signals that form a “glyco-immune checkpoint” and dampen antitumor immunity (Lin et al., 2025; Zhang et al., 2025). Thirdly, interventional desialylation, achieved through antibody-sialidase conjugates, can effectively strip sialoglycans from the tumor surface. This ablates the sialoglycan-Siglec interactions, thereby promoting the activation and polarization of immune cells (particularly macrophages) and enhancing the efficacy of immune checkpoint blockade (ICB) therapy in *in vivo* mouse models (Stanczak et al., 2022). This approach holds substantial value for advancing clinical cancer treatment strategies.

### 3.5 Neurodegenerative diseases: sialidase involvement in disrupted neuroprotective mechanisms

Sialidases modulate sialylation on neuronal and myelin surfaces as well as the composition of brain gangliosides, thereby participating in the maintenance of neurological homeostasis. For instance, dysregulation or loss of activity in NEU1, NEU3, or NEU4 may contribute to neurodegenerative changes via multiple pathways.

#### 3.5.1 NEU1 regulates lysosomal APP processing and Aβ generation: desialylation imbalance drives amyloid pathology

One of the central pathological hallmarks of Alzheimer’s disease (AD) is the deposition of amyloid-β (Aβ) plaques (Zheng and Wang, 2025). Conventional understanding has predominantly focused on the processing and secretion of amyloid precursor protein (APP) via the endocytic-Golgi-plasma membrane trafficking pathway involving secretase cleavage (Hur, 2022; Wang et al., 2024a; Wang et al., 2024b). Concurrently, some researchers propose that the lysosomal pathway, particularly lysosomal exocytosis, may constitute an overlooked yet significant “exit” route for Aβ (Im et al., 2023; Tsang et al., 2025). Under these conditions, deficiency or reduced function of NEU1-the primary lysosomal sialidase-can lead to excessive sialylation of APP within lysosomes, diminished degradation efficiency, and aberrant lysosomal exocytosis. These disruptions collectively increase extracellular Aβ release and plaque formation (Annunziata et al., 2013). For instance, in animal models, Neu1 deficiency results in Aβ-like accumulation in the mouse brain, whereas delivery of NEU1 via adeno-associated virus (AAV) into an AD animal model significantly reduces amyloid-β plaques (Annunziata et al., 2013). In summary, these observations reveal that the NEU1-lysosome axis may play a dual role in AD pathology, acting both as a direct pathogenic factor and, under certain conditions, exerting protective effects.

#### 3.5.2 NEU3-mediated desialylation of the glycocalyx: promoting microglia-mediated non-contact synaptic pruning and network destabilization

Recent studies have revealed that activated microglia release extracellular vesicles (EVs) enriched with NEU3 and deliver them to the surface of neighboring neurons (Delaveris et al., 2023; Cheng et al., 2025). In this context, NEU3 hydrolyzes sialylated gangliosides and glycolipids on the neuronal membrane, leading to desialylation and disruption of the membrane glycocalyx. This results in significant alterations in the composition and structure of the neuronal glycocalyx (Delaveris et al., 2023). In experimental models, NEU3-induced desialylation of neurons reduces network synchrony and weakens connectivity, whereas inhibition or deficiency of NEU3 prevents this effect, indicating a crucial role for the desialylating activity of NEU3 in this process (Delaveris et al., 2023).

This discovery offers a novel perspective for understanding neurodegenerative diseases. For instance, in Alzheimer’s disease, Parkinson’s disease, and other conditions accompanied by neuroinflammation, microglial activation is a common pathological feature (Chen et al., 2025; Erdag and Haskologlu, 2025). Persistent abnormal release or overactivation of NEU3 may lead to chronic synaptic desialylation and “over-pruning”, which could further accelerate network destabilization, cognitive decline, and neurodegeneration (Delaveris et al., 2023; Cheng et al., 2025; Lara de Deus et al., 2025).

In summary, NEU3 is increasingly being recognized as a potential therapeutic target in neuroinflammatory and neurodegenerative conditions (Schengrund, 2025). Accordingly, strategies aimed at inhibiting NEU3 activity or preserving the integrity of the neuronal glycocalyx may confer neuroprotective benefits (Delaveris et al., 2023; Cheng et al., 2025).

#### 3.5.3 NEU3-NEU4 co-regulation of brain ganglioside homeostasis: dual-enzyme deficiency leads to neuroinflammation, synaptic lipid disruption, and behavioral deficits

NEU3 and NEU4 are the predominant sialidases responsible for the catabolism of sialylated gangliosides in the central nervous system. Research in this field has, for the first time, utilized NEU3/NEU4 double-knockout (DKO) mice to demonstrate the crucial role of these enzymes in maintaining ganglioside homeostasis in the brain *in vivo (*Pan et al., 2017; Allende et al., 2023). Compared to wild-type controls, DKO mice exhibited significant accumulation of GM3 gangliosides in both neurons and glial cells, along with a marked reduction in membrane-associated GM1 ganglioside levels (Pan et al., 2017).

Specifically, in this study, NEU3/NEU4 DKO mice displayed prominent neuroinflammatory features at the histological level, including microgliosis, astrogliosis, and lipofuscin deposition. At the cellular functional level, primary cultured neurons from these mice showed impaired axonal and dendritic growth. Furthermore, behavioral analyses revealed that the DKO mice exhibited significant learning and memory deficits (Pan et al., 2017).

Therefore, this study demonstrates that the balanced activity of NEU3 and NEU4 is essential for regulating ganglioside distribution, thereby maintaining cerebral lipid homeostasis. Disruption of this balance may drive a cascade of events including neuroinflammation, synaptic dysfunction, and cognitive decline. This finding provides important evidence linking sialidase function to neurodegenerative pathology.

#### 3.5.4 The sialylation-SIGLEC regulatory axis: a core immune mechanism connecting sialidase activity to neuroinflammation

Current evidence suggests that sialylated glycans on the neuronal surface engage with Siglec receptors (such as CD33 and Siglec-11) on microglia, transmitting an “inhibitory recognition” signal (Gonzalez-Gil et al., 2022; Wißfeld et al., 2024; Cheng et al., 2025). This interaction helps maintain microglial homeostasis by suppressing excessive phagocytosis and inflammatory responses (Wißfeld et al., 2024). For instance, sialidase-mediated desialylation can attenuate this inhibitory pathway, thereby priming microglia to adopt a more pro-inflammatory or phagocytic phenotype, which may subsequently contribute to synapse loss and neurodegeneration (Puigdellívol et al., 2020). *In vitro* studies have demonstrated that modulating sialylation levels or Siglec signaling can alter microglial phenotype and influence the processing of pathogenic proteins such as Aβ and α-synuclein (Puigdellívol et al., 2020). Furthermore, human genetic studies have shown that the *CD33* locus, encoding an inhibitory Siglec receptor, is significantly associated with Alzheimer’s disease risk, providing strong epidemiological and genetic support for this mechanism (Tu et al., 2024).

Nevertheless, the existing evidence primarily consists of independent experiments and reviews. There remains a lack of direct, *in vivo* causal evidence that fully connects the cascade from “sialidase dysregulation → altered neuronal sialylation → impaired SIGLEC recognition → microglial phenotypic shift → consequent neurological and behavioral deficits.” Addressing this gap represents a critical focus for future research.

#### 3.5.5 Clinical opportunities of sialidases: diagnostic and therapeutic applications

Sialidases (neuraminidases; primarily NEU1-NEU4 in humans) influence key biological processes such as receptor activation, immune recognition, cell adhesion, and metabolism by removing terminal sialic acid residues from glycoproteins and glycolipids, thereby altering the cell surface glycosylation phenotype (Du et al., 2024; Zhu et al., 2024; Schengrund, 2025). Consequently, due to their functional roles in various diseases-including cancer, neurodegenerative disorders, chronic inflammation, and infections-alterations in sialidase expression levels and enzymatic activity are increasingly being explored as potential diagnostic and prognostic biomarkers, as well as proposed as novel therapeutic intervention targets (Lillehoj et al., 2022; Du et al., 2024; Mei et al., 2024; Fremuth et al., 2025; Schengrund, 2025). Therefore, this section will review current research progress, representative evidence, and future challenges in the fields of diagnostic applications and therapeutic development.

3.3 Sialidases as Diagnostic and Prognostic BiomarkersThe expression and activity of sialidases, particularly NEU1 and NEU3, in tissues or body fluids exhibit variations under multiple pathological conditions and have been associated with disease progression or prognosis in some studies, indicating their potential as biomarkers (Aljohani et al., 2024; Du et al., 2024; Schengrund, 2025). Firstly, numerous tumor histological and transcriptomic studies have shown that NEU1 or NEU3 expression is upregulated in several solid tumors (e.g., melanoma, pancreatic cancer, bladder cancer), and their high expression is often correlated with increased invasiveness and poor prognosis (Peng et al., 2022; Du et al., 2024; Tatsuta et al., 2024). Secondly, studies in neurodegenerative diseases and certain inflammatory disorders have shown that alterations in desialylation- or sialylation-related glycan profiles in plasma or cerebrospinal fluid are associated with disease pathology, progression, or inflammatory status (Furukawa et al., 2024; Krüger et al., 2024; Onigbinde et al., 2025b). These findings suggest that desialylation-related molecules could serve as candidate indicators for monitoring disease activity or immune status in selected conditions (Furukawa et al., 2024; Krüger et al., 2024; Onigbinde et al., 2025b; Onigbinde et al., 2025a). However, current evidence largely originates from small-scale cohort studies, and their clinical utility requires further validation with larger sample sizes and under standardized conditions.

Regarding diagnostic methods, current research primarily employs two complementary categories of techniques (Gattani et al., 2023; Onigbinde et al., 2025a). The first category comprises enzyme activity assays, which use fluorescent or colorimetric substrates to assess sialidase catalytic activity in biological samples through measurable signals generated by enzymatic cleavage of sialylated substrates (Gattani et al., 2023; Yang et al., 2024). The second category comprises glycomic analysis, including mass spectrometry-based glycan profiling and glycan array technology, which characterizes glycosylation changes in biological samples such as plasma and tissue to indirectly reflect sialylation status, including features such as terminal galactose exposure or reduced polysialylation (Kurogochi et al., 2025; Onigbinde et al., 2025a).

From a methodological perspective, enzyme activity assays and glycomic analysis are fundamentally different when studying sialidase function (Alvarado-Melendez et al., 2025; Kurogochi et al., 2025; Onigbinde et al., 2025a). Enzyme activity assays, which provide exogenous sialylated substrates and detect their hydrolysis products, currently represent the only method capable of directly assessing the catalytic activity of sialidases (Alvarado-Melendez et al., 2025; Smirnov et al., 2025). In contrast, glycomic analysis reflects the cumulative outcome of desialylation by profiling the overall changes in glycan structures and sialic acid modification states within biological samples but cannot be directly equated with enzyme activity measurement (Kurogochi et al., 2025; Onigbinde et al., 2025a). Since glycan profile alterations are co-regulated by multiple factors including sialidases, sialyltransferases, and glycan metabolism, glycomic results typically require integration with enzyme activity data or genetic evidence to infer the role of sialidases in pathological processes (Togayachi et al., 2026).

Although existing small-scale cohort and translational studies provide some support for the aforementioned candidate biomarkers and signals, further steps are necessary to advance them towards clinical application. These include the standardization of detection methods, validation across multiple centers, and combined evaluation with established clinical parameters (Kauskot et al., 2025; Kurogochi et al., 2025).

### 3.6 Sialidases as therapeutic targets: strategies and recent advances

Due to the pivotal roles of the sialidase family in pathological processes such as infection, inflammation, tumor progression, and fibrosis, these enzymes have gradually emerged as highly attractive therapeutic targets (Du et al., 2024; Schengrund, 2025). At present, therapeutic strategies centered on sialidases and their related glycan pathways can be broadly classified into three categories. The first involves direct modulation of sialidase activity, including the classical inhibitors developed against viral neuraminidases as well as selective inhibitors targeting human NEU1-NEU4 isoenzymes (Keil et al., 2022; Schengrund, 2025). The second aims to restore immune cell activity by interfering with the sialylation-Siglec inhibitory axis (Wieboldt et al., 2024; Bashian et al., 2026). The third seeks to achieve more precise local regulation of desialylation through exosomes, nanovesicles, or engineered enzyme-delivery systems (Ansun Biopharma, Inc, 2024; Sharma et al., 2024). Notably, although sialidase-targeted therapies have shown promising translational potential, the maturity of different strategies varies substantially. For example, the development and application of drugs targeting viral neuraminidases have already reached a relatively systematic stage, whereas selective inhibitors targeting human sialidase isoforms remain largely in the preclinical phase (Keil et al., 2022; Ansun Biopharma, Inc, 2024). By contrast, glycan-editing strategies mediated by engineered sialidases have already begun to enter early-stage clinical investigation (Sharma et al., 2024; Bashian et al., 2026). The mechanisms involved are summarized as follows.

First, with regard to direct inhibition, influenza viral neuraminidase was among the earliest sialidase targets to be successfully translated into clinical antiviral therapy (Keil et al., 2022; Sassine et al., 2024). Neuraminidase inhibitors such as oseltamivir and zanamivir effectively block the desialylation activity of viral neuraminidase, thereby inhibiting the release of viral particles from the host cell surface and reducing viral spread; accordingly, they are now widely used in the treatment of influenza (Winquist et al., 1999; Gitto et al., 2025). In contrast, the development of drugs targeting human sialidases is still at an early stage (Keil et al., 2022). Nevertheless, with continued progress in this field, several selective inhibitors have shown therapeutic potential in disease models in recent years. For example, the NEU1-selective inhibitors C9-BA-DANA and CG33300/CG33301 have demonstrated beneficial effects in bleomycin-induced mouse models of pulmonary fibrosis, including attenuation of inflammatory responses, reduction of collagen deposition, and amelioration of fibrosis, suggesting that NEU1 may represent an important therapeutic target in fibrotic and inflammatory diseases (Luzina et al., 2021; Du et al., 2024). In addition, selective DANA derivatives targeting NEU3 can alleviate the progression of pulmonary fibrosis by inhibiting desialylation-related activation of TGF-β1 (Karhadkar et al., 2021). Overall, however, because publicly available information has not shown that selective small-molecule inhibitors targeting human NEU1 or NEU3 have entered clearly defined phase I-III registered clinical trials, this direction as a whole remains at the stage of preclinical validation.

Second, research on strategies targeting the sialylation-Siglec axis has advanced rapidly in recent years (Pinho et al., 2025; Zhang et al., 2025). By blocking hypersialylation on tumor cells or other pathological cells, or by directly interrupting inhibitory Siglec signaling, investigators have been able to weaken sialic acid-Siglec-mediated immunosuppression within tumors or other pathological microenvironments, thereby restoring or enhancing the effector functions of immune cells (Boelaars and Kooyk, 2024; Wieboldt et al., 2024; Lin et al., 2025). For example, engineered sialidase-based strategies have already begun to enter clinical application (Bashian et al., 2026). E-602, an engineered human sialidase fusion protein, removes immunosuppressive sialoglycans from the surfaces of tumor cells and immune cells, thereby enhancing antitumor immunity (Sharma et al., 2024). It is currently being investigated in the phase I/II GLIMMER-01 study in patients with advanced solid tumors (Palleon Pharmaceuticals, Inc, 2025). Taken together, these findings indicate that therapies targeting the sialylation-Siglec axis are gradually transitioning from proof-of-concept studies to early clinical exploration (Pinho et al., 2025; Zhang et al., 2025).

Third, in recent years, increasing attention has been given to exosome- and nanovesicle-based delivery systems, as well as other engineered platforms, for achieving more precise local modulation of desialylation (Yang et al., 2024; Liu et al., 2025). For instance, by conjugating sialidases to tumor-targeting antibodies, these enzymes can be preferentially enriched on the tumor cell surface and locally remove sialic acids, thereby enhancing local immune-mediated killing while reducing systemic adverse effects (Stanczak et al., 2022; Wu et al., 2023). This concept may also have broader relevance in other immune-related settings, including inflammatory and autoimmune diseases (Tvaroška, 2025). Beyond oncology, another representative strategy that has entered the clinical stage is DAS181 (Ansun Biopharma, Inc, 2024). DAS181 is essentially a recombinant sialidase fusion protein rather than a sialidase inhibitor; it acts by removing sialic acid receptors from the surface of host respiratory epithelial cells, thereby blocking the attachment and invasion of influenza and parainfluenza viruses (Chemaly et al., 2021). To date, DAS181 has completed multiple early studies and has advanced to phase III clinical development for parainfluenza virus lower respiratory tract infection in immunocompromised patients (Ansun Biopharma, Inc, 2024). Overall, the clinical translation of sialidase-related therapies is not limited to the conventional path of small-molecule inhibitors, but also includes emerging therapeutic modalities based on engineered enzymes for glycan editing and local microenvironment remodeling (Zhang et al., 2025; Bashian et al., 2026). However, exosome- and nanovesicle-based delivery strategies as a whole remain at an early stage of development, and the currently available evidence is still derived mainly from preclinical studies (Yang et al., 2024; Li et al., 2025).

To more clearly illustrate the translational progress in this field, **Table 3** summarizes representative sialidase-targeted drugs and therapeutic strategies according to their current stage of development.

**Table 3:** Representative sialidase-targeting drugs/strategies at different stages of development.

Drug/Strategy	Main target/intended target	Indication(s)	Mechanism of action	Development stage
Oseltamivir; Zanamivir (Hayden et al., 1997; Centers for Disease Control and Prevention (CDC), 2026)	Viral neuraminidase	Influenza	Inhibit influenza viral neuraminidase activity, thereby blocking viral release and spread	Marketed
C9-BA-DANA (Hyun et al., 2016)	Human NEU1	Pulmonary fibrosis, etc.	Selectively inhibits NEU1 and alleviates desialylation-related inflammation and fibrotic responses	Preclinical
CG33300/CG33301 (Guo et al., 2018b; Luzina et al., 2021)	Human NEU1	Pulmonary fibrosis, etc.	Selectively inhibits NEU1 and reduces pulmonary inflammation and collagen deposition	Preclinical
NEU3-selective DANA derivatives (Guo et al., 2018a; Karhadkar et al., 2021)	Human NEU3	Pulmonary fibrosis	Inhibit NEU3-mediated desialylation and TGF-β1 activation	Preclinical
E-602 (Sharma et al., 2024; Palleon Pharmaceuticals, Inc, 2025)	Surface sialoglycans on tumor and immune cells	Advanced solid tumors	An engineered human sialidase fusion protein that removes immunosuppressive sialoglycans and enhances antitumor immunity	Phase I/II
DAS181 (Fludase) (Zenilman et al., 2015; Ansun Biopharma, Inc, 2024)	Sialic acid receptors on the host respiratory epithelium	Influenza/parainfluenza virus infection	A recombinant sialidase fusion protein that removes host-cell surface sialic acid receptors, thereby blocking viral attachment and entry	Phase III; multiple early-phase clinical studies completed

## 4 Summary and outlook

Sialidase, a master regulator in glycobiology, orchestrates critical biological processes-intercellular communication, receptor signaling, immune recognition, and pathogen adhesion-through precise removal of terminal sialic acid residues from glycoproteins, glycolipids, and polysaccharides (Du et al., 2024; Zhu et al., 2024; Schengrund, 2025). Two decades of systematic investigation into its classification, nomenclature, catalytic mechanisms, and structural architecture have cemented a robust foundation for decoding its molecular functions and pathological roles (Keil et al., 2022; Zhu et al., 2024). Emerging evidence now solidifies its pathogenic significance across hereditary disorders, infectious diseases, inflammatory/autoimmune conditions, cancer, and neurodegenerative diseases, positioning it as a unifying molecular link between physiological homeostasis and disease pathogenesis (Lillehoj et al., 2022; Cheng et al., 2025; Schengrund, 2025).

The translational trajectory of sialidase is undergoing a paradigm shift-from a basic research cornerstone to a clinical innovation engine. Expression dynamics and activity profiles of isoforms such as NEU1 and NEU3 now serve as molecular barometers for tumor aggressiveness, inflammatory activity, and neurological disorder severity, enabling molecular subtyping and prognostic stratification. Concurrently, selective inhibitors, monoclonal antibodies, and glycan-engineering strategies have demonstrated therapeutic efficacy in preclinical models and early-phase trials, particularly in cancer immunotherapy and chronic inflammation modulation.

However, translational hurdles persist. The structural conservation among isoforms complicates selective targeting, while the dual engagement in physiological and pathological processes necessitates balanced intervention strategies to avoid off-target effects. Dynamic regulatory networks across tissues, disease stages, and pathological contexts remain incompletely mapped, constraining precision intervention design. Critical translational steps-method standardization, multicenter validation, and biomarker integration-require sustained focus.

Looking ahead, technological revolutions in glycomics, single-cell omics, cryo-EM, and structural biology promise unprecedented spatial-temporal resolution of sialidase functional maps, enabling precise structure-function decoding (Keisham and Tateno, 2024; Mycroft-West et al., 2025; Onigbinde et al., 2025a; Seo et al., 2025). Integration with AI-driven drug discovery, ultra-sensitive detection platforms, and multi-omics data fusion will transform sialidase from a “pathology-associated molecule” into a clinically actionable diagnostic and therapeutic target (Seo et al., 2025; Zhang et al., 2025). This evolution will not only deepen glycobiology-disease mechanism synergies but may also pioneer novel prevention and treatment paradigms in oncology, inflammation, and neurodegeneration-ushering in a new era of precision glycotherapeutics.
